# Parental Perceptions of Tennessee’s Mature Minor Doctrine

**DOI:** 10.1001/jamanetworkopen.2025.5798

**Published:** 2025-04-18

**Authors:** Sarah F. Loch, Elizabeth McNeer, Michelle Fiscus, William D. Dupont, Stephen W. Patrick

**Affiliations:** 1 Department of Health Policy and Management, Emory University Rollins School of Public Health, Atlanta, Georgia; 2 Department of Biostatistics, Vanderbilt University Medical Center, Nashville, Tennessee; 3 Fiscus Health Consulting, Alexandria, Virginia; 4 Department of Pediatrics, Emory University, Atlanta, Georgia; 5 Health Services Research Center, Emory University, Atlanta, Georgia

## Abstract

This survey study evaluates whether the wording of questions regarding mature minor doctrines is associated with parental opinions about adolescent consent for medical services overall and for specific care types.

## Introduction

Mature minor doctrines (MMDs) permit adolescent consent for medical services in some states.^1^ Tennessee’s longstanding MMD was challenged during the COVID-19 pandemic by state lawmakers concerned for children obtaining COVID-19 vaccines against parental wishes.^2^ Tennessee’s 2023 Mature Minor Clarification Act (MMCA) was passed in response, counteracting the MMD and requiring parental consent for childhood vaccines and written consent for COVID-19 vaccines,^3^ fueling concern for childhood vaccination rates.^2^

Although most US individuals believe childhood vaccines are safe, those considering them extremely or very important decreased 25 percentage points between 2001 and 2024,^4^ and support of school vaccine requirements has decreased since 2019, a possible response to increasing opposition of government mandates.^5^ Whether MMCA reflects parental opinion is unclear. We used a randomized survey experiment to evaluate whether framing questions about MMD was associated with parental opinions of adolescent consent for medical services overall and for specific care types.

## Methods

In this survey study, Ipsos online KnowledgePanel and external opt-in was used to survey a population representative sample of Tennessee parents with at least 1 child younger than 18 years in their household between October 19 and November 28, 2021 (eAppendix 1 in Supplement 1). Demographics including race and ethnicity were self-reported using panel classifications. Data on race and ethnicity were collected to evaluate issues of equity. This study followed AAPOR reporting guidelines and best practices and was exempt by the institutional review board of Vanderbilt University Medical Center. Participation in Ipsos surveys is voluntary, and survey-specific consent was not required by Ipsos or the institutional review board.

Respondents were randomly assigned vignettes describing adolescent consent (aged 14-18 years) using the terms (1) *mature minor doctrine* or (2) *rule* in place of *mature minor doctrine* with an example (eAppendix 2 in Supplement 1). Respondents rated their support or opposition of adolescent consent using a 5-point Likert scale and indicated agreement or disagreement for application to the following care types: (1) mental health services, (2) reproductive health services, and (3) preventive care.

Survey weights based on 2019 American Community Survey for Tennessee adults were applied (eAppendix 1 in Supplement 1), and vignette groups were compared using a Rao-Scott corrected χ^2^ test. Two-sided *P* < .05 was considered statistically significant. Analyses were done from March to November 2024 with the survey package in R version 4.2.1 (R Project for Statistical Computing).

## Results

Weighted characteristics of the MMD (n = 506; mean age, 38.7 years [95% CI, 37.4-39.9 years]; 361 female, unweighted number [58.9%]) and rule (n = 520, mean age, 38.7 years [95% CI, 37.4-40.0 years]; 357 female, unweighted number [55.1%]) groups were similar (Table). The survey had an 86.9% qualification rate and 1026 responses after exclusions for quality reasons (eAppendix 1 in Supplement 1). There was a 20.3 percentage point difference in support of adolescent consent between the groups, with 22.9% (95% CI, 18.0%-27.8%) of the MMD group in support compared with 43.2% (95% CI, 36.9%-49.5%) of the rule group (*P* < .001) (Figure).

**Table.** Characteristics of Parents Randomized to Receive Mature Minor Doctrine and Rule Vignettes

Characteristic	Unweighted No. (weighted %) [95% CI]	
	Mature minor doctrine (n = 506)	Rule (n = 520)
No. of children in household aged <18 y		
1	238 (49.2) [42.9-55.5]	203 (42.8) [36.4-49.2]
2	159 (32.4) [26.4-38.4]	196 (34.8) [29.1-40.5]
≥3	109 (18.4) [14.1-22.8]	121 (22.4) [17.4-27.3]
Age, weighted mean (95% CI), y	38.7 [37.4-39.9]	38.7 [37.4-40.0]
Gender		
Female	361 (58.9) [52.5-65.3]	357 (55.1) [48.8-61.5]
Male	145 (41.1) [34.7-47.6]	163 (44.9) [38.5-51.2]
Education		
Less than high school	22 (3.2) [1.6-4.7]	23 (3.1) [1.6-4.7]
High school	129 (32.3) [26.1-38.6]	134 (30.9) [24.9-37.0]
Some college	170 (29.3) [23.7-34.9]	164 (31.7) [25.9-37.5]
Bachelor’s degree or higher	185 (35.3) [29.3-41.2]	199 (34.3) [28.5-40.1]
Race and ethnicity		
Black non-Hispanic	53 (15.4) [10.7-20.1]	64 (17.1) [12.1-22.2]
White non-Hispanic	393 (72.3) [66.5-78.2]	397 (73.5) [67.8-79.1]
Other^a^	60 (12.3) [8.0-16.6]	59 (9.4) [6.0-12.9]
Tennessee region		
East	215 (38.6) [32.6-44.7]	228 (40.0) [34.0-46.0]
Middle	183 (39.9) [33.6-46.1]	185 (38.9) [32.7-45.1]
West	108 (21.5) [16.3-26.7]	106 (21.1) [15.9-26.3]
Marital status		
Married	325 (71.8) [66.4-77.3]	326 (68.6) [62.9-74.3]
Unmarried	181 (28.2) [22.7-33.7]	194 (31.4) [25.8-37.1]
Annual household income, $		
<25 000	121 (11.1) [8.1-14.0]	125 (10.1) [7.8-12.4]
25 000-49 999	121 (16.4) [12.4-20.3]	141 (23.4) [18.5-28.2]
50 000-99 999	166 (37.3) [31.3-43.3]	150 (33.7) [27.7-39.7]
≥100 000	98 (35.3) [28.7-41.8]	104 (32.8) [26.5-39.2]
Employment status		
Working	499 (97.6) [95.3-99.9]	507 (96.3) [93.7-98.9]
Not working	7 (2.4) [0.1-4.7]	13 (3.7) [1.2-6.3]
Homeowner status		
Owned or being bought by you or someone in your household	330 (73.5) [68.3-78.8]	341 (71.0) [65.6-76.3]
Rented for cash	158 (23.6) [18.7-28.6]	158 (26.0) [20.9-31.1]
Occupied without payment of rent	18 (2.9) [0.7-5.1]	21 (3.0) [0.9-5.2]
Type of home		
Single-family home	396 (81.3) [76.3-86.3]	419 (84.4) [80.3-88.6]
Apartment building	70 (11.2) [7.3-15.1]	61 (10.5) [6.7-14.3]
Other (eg, mobile home, boat, van, recreational vehicle)	40 (7.5) [4.0-11.0]	40 (5.1) [3.1-7.0]

^a^ Other includes any other non-Hispanic race, Hispanic, and 2 or more non-Hispanic races. These categories were grouped owing to small numbers within each classification.

**Figure.**
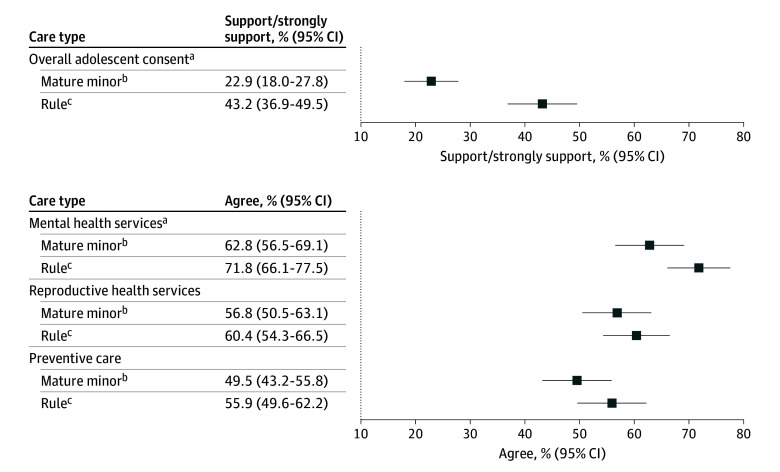
Support of Adolescent Consent for Health Care and Agreement for Specific Care Types Among Mature Minor and Rule Vignette Groups See eAppendix 2 in Supplement 1 for wording of the vignettes. ^a^*P* < .05. ^b^Two individuals in the mature minor group refused to answer for all health care services and were excluded from the percentage calculations (n = 504). ^c^One individual in the rule group refused to answer for reproductive health services and was excluded from the percentage calculations (n = 519).

The rule group was more likely to agree that mental health services should be allowable with adolescent consent (71.8%; 95% CI, 66.1%-77.5%) compared with the MMD group (62.8%; 95% CI, 56.5%-69.1%) (*P* = .04) (Figure). Framing did not influence agreement for reproductive health care or preventive care.

## Discussion

In this survey study, parents were less supportive of adolescent consent when framed using MMD without further clarification, mirroring media portrayal of MMD at the time of the survey. In the context of a single state, this exemplifies the imperative to account for the influence of current culture like the growing parental rights movement on health care decision-making and the urgency of trustworthy health messaging from clinicians and public health agencies.

Since MMCA was passed, the 2024 Families’ Rights and Responsibilities Act^6^ was enacted, which broadly requires parental consent for adolescent physical and mental health care. Our results suggest the trend of parental rights legislation may not align with parents’ attitudes. Taking into account possible response bias common in surveys, we found a near or clear majority agreed with adolescent consent for preventive, reproductive, and mental health care. Taken together, the need for legislation that protects adolescent access to specific services would be in line with parental opinion and should be considered.

## Notes

Supplement 1. **eAppendix 1.** Survey methods **eAppendix 2.** Mature minor and rule vignettes

Supplement 2.
Data Sharing Statement
