# Supplementary material for: Parental Perceptions of Tennessee’s Mature Minor Doctrine
Source: JAMA Netw Open. 2025 Apr 18;8(4):e255798. doi: 10.1001/jamanetworkopen.2025.5798 (PMC12008757; doi:10.1001/jamanetworkopen.2025.5798)
Supplement: Supplement 1. — eAppendix 1. Survey methods eAppendix 2. Mature minor and rule vignettes [file jamanetwopen-e255798-s001.pdf]

Supplemental Online Content

Loch SF, McNeer E, Fiscus M, Dupont WD, Patrick SW. Parental perceptions of Tennessee's mature minor doctrine. *JAMA Netw Open*. 2025;8(4):e255789. doi:10.1001/jamanetworkopen.2025.5798

eAppendix 1. Survey methods

eAppendix 2. Mature minor and rule vignettes

This supplemental material has been provided by the authors to give readers additional information about their work.

© 2025 Loch SF et al. *JAMA Network Open*.

eAppendix 1. Survey methods

Ipsos Public Affairs KnowledgePanel is an existing online panel of households assembled by probability-based address sampling and external opt-in. Ipsos offers internet access and devices as necessary for participation in KnowledgePanel and provides incentives for panel participation.

Participants were selected using stratified random sampling. Email invitations were sent with periodic reminders for non-responders. Survey responses were not included in the qualified sample for saying no/refusing parent status, saying no/refusing to identify the number of children in their household, refusing or providing their own age as 17 or younger, or not residing in Tennessee. Qualified completions were dropped from the final sample for skipping 50% or more of eligible questions or finishing survey in 25% or less time than the median completion time (i.e., speeding). The survey was offered in English. The sequence in which respondents were presented with care types (reproductive health services, mental health services, preventive care) to indicate agreement or disagreement was randomized. In addition to questions about the mature minor doctrine, participants responded to other topics about the health and wellbeing of their children. The survey had a median completion time of 12.6 minutes.

Survey weights based on distributions from the 2019 American Community Survey for Tennessee adults age 18 years and older with child(ren) in their household aged 0-17 years were developed to achieve population-weighted estimates using geodemographic benchmarks for race/ethnicity (White, Black, other, Hispanic, two or more races), gender (male, female) by age (18-34, 35-44, ≥45 years), gender (male, female) by race/ethnicity (White, Black, all others), age (18-34, 35-44, ≥45 years) by race/ethnicity (White, Black, all others), education (less than high school or high school, some college, bachelor's degree or higher) by race/ethnicity (White, Black, all others), household income (<$25k, $25k-49,999, $50k-74,999, ≥$75k) by race/ethnicity (White, Black, all others), and Tennessee region (east, middle, west).

eAppendix 2. Mature minor and rule vignettes

The mature minor vignette question was worded as follows: "In Tennessee, the 'mature minor' doctrine allows health care providers to treat children ages 14-18 without their parent's consent if the health care provider determines the child is sufficiently mature to make their own health care decisions. How much do you support or oppose the 'mature minor' doctrine? The rule vignette question was worded as follows: "In Tennessee, there is a rule that allows health care providers to treat children ages 14-18 without their parent's consent if the health care provider determines the child is sufficiently mature to make their own health care decisions. This permits, for example, 17 year old college students to obtain preventive services like meningitis vaccines while at college. How much do you support or oppose this rule?" The care type vignette question was worded as follows: "Do you agree or disagree that the ['mature minor doctrine'/rule] that allows health care providers to treat children ages 14-18 without their parent's consent if the child is sufficiently mature should be used in the following services? Mental health services (for example: talking to a therapist); Reproductive health services (for example: obtaining prescription birth control, but not abortion); Preventive care (for example: getting shots)."

© 2025 Loch SF et al. *JAMA Network Open*.
