# Supplementary material for: Parental Perceptions of Tennessee’s Mature Minor Doctrine
Source: JAMA Netw Open. 2025 Apr 18;8(4):e255798. doi: 10.1001/jamanetworkopen.2025.5798 (PMC12008757; doi:10.1001/jamanetworkopen.2025.5798)
Supplement: Supplement 2. — Data Sharing Statement [file jamanetwopen-e255798-s002.pdf]

Data Sharing Statement

Loch. Parental Perceptions of Tennessee's Mature Minor Doctrine. *JAMA Netw Open*. Published April 18, 2025. doi:10.1001/jamanetworkopen.2025.5798

Data
Data available: Yes
Data types: Deidentified participant data, Data dictionary
How to access data: stephen.patrick@emory.edu
When available: With publication

Supporting Documents
Document types: Statistical/analytic code
How to access documents: stephen.patrick@emory.edu
When available: With publication

Additional Information
Who can access the data: researchers whose proposed use of the data has been approved
Types of analyses: for any purpose
Mechanisms of data availability: after approval of a proposal
Any additional restrictions: none
